# Temple Monkeys and Health Implications of Commensalism, Kathmandu, Nepal

**DOI:** 10.3201/eid1206.060030

**Published:** 2006-06

**Authors:** Lisa Jones-Engel, Gregory A. Engel, John Heidrich, Mukesh Chalise, Narayan Poudel, Raphael Viscidi, Peter A. Barry, Jonathan S. Allan, Richard Grant, Randy Kyes

**Affiliations:** * University of Washington, Seattle, Washington, USA;; † Swedish Providence Family Medicine Residency, Seattle, Washington, USA;; ‡ University of New Mexico Medical School, Albuquerque, New Mexico, USA;; § Tribhuvan University, Kathmandu, Nepal;; ¶ Nepal Biodiversity Research Society, Kathmandu, Nepal;; # Department of National Park and Wildlife Conservation, Kathmandu, Nepal;; ** Johns Hopkins University, Baltimore, Maryland, USA;; †† University of California, Davis, California, USA;; ‡‡ Southwest Foundation for Biomedical Research, San Antonio, Texas, USA

**Keywords:** simian retrovirus, simian T-cell lymphotropic virus, Cercopithecine herpesvirus 1, simian foamy virus, SV40, RhCMV, Macaca, primate zoonoses, Asia, temple monkeys, Nepal

## Abstract

The threat of zoonotic transmission of infectious agents at monkey temples highlights the necessity of investigating the prevalence of enzootic infectious agents in these primate populations. Biological samples were collected from 39 rhesus macaques at the Swoyambhu Temple and tested by enzyme-linked immunosorbent assay, Western blot, polymerase chain reaction, or combination of these tests for evidence of infection with rhesus cytomegalovirus (RhCMV), *Cercopithecine herpesvirus* 1 (CHV-1), simian virus 40 (SV40), simian retrovirus (SRV), simian T-cell lymphotropic virus (STLV), simian immunodeficiency virus (SIV), and simian foamy virus (SFV). Antibody seroprevalence was 94.9% to RhCMV (37/39), 89.7% to SV40 (35/39), 64.1% to CHV-1 (25/39), and 97.4% to SFV (38/39). Humans who come into contact with macaques at Swoyambhu risk exposure to enzootic primateborne viruses. We discuss implications for public health and primate management strategies that would reduce contact between humans and primates.

## Abstract 2

Humans in contact with macaques risk exposure to enzootic primateborne viruses.

Most pathogens that affect humans are thought to have originated in animals and subsequently evolved to successfully parasitize human populations (*1*). Proximity and physical contact between animals and humans provide the opportunity for infectious agents to pass between the groups. Whether a particular infectious agent can successfully make the cross-species jump depends in part on the new host environment (*1*). By virtue of their genetic, physiologic, and behavioral similarity to humans, nonhuman primates (hereafter referred to as primates) are particularly likely sources of emerging infectious agents with the capacity to infect humans, and primate-to-human cross-species transmission of infectious agents has become a focus of scientific inquiry. Because human-primate contact is common in Asia, this continent is a rich area in which to pursue this research. We examine the prevalence of selected enzootic primateborne viruses in a population of rhesus macaques (*Macaca mulatta*) that lives in close proximity to humans.

## Monkey Temple Context for Cross-species Transmission

Monkey temples can be found throughout South and Southeast Asia, where primates play a role in Hindu and Buddhist culture (*2*). Macaque species, because they can thrive in human-altered environments, are the primates most often associated with temples. Extensive, unregulated, and often close contact between humans and primates occurs at these sites (*3*). Persons who live or work in or around monkey temples are among those who frequently come into contact with temple monkeys (*4*). Other persons may come into contact with temple macaques when they visit for purposes of worship, recreation, or tourism. Worldwide, monkey temples may account for more human-primate contact than any other context (*5*).

**Figure 1:**
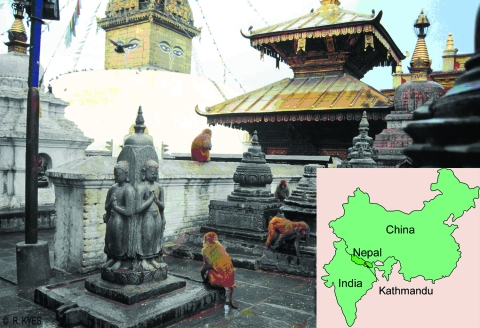
Swoyambhu Temple in Kathmandu, Nepal, is home to ≈400 free-ranging rhesus macaques (*Macaca mulatta*). (Photo by R. Kyes.)

**Figure 2:**
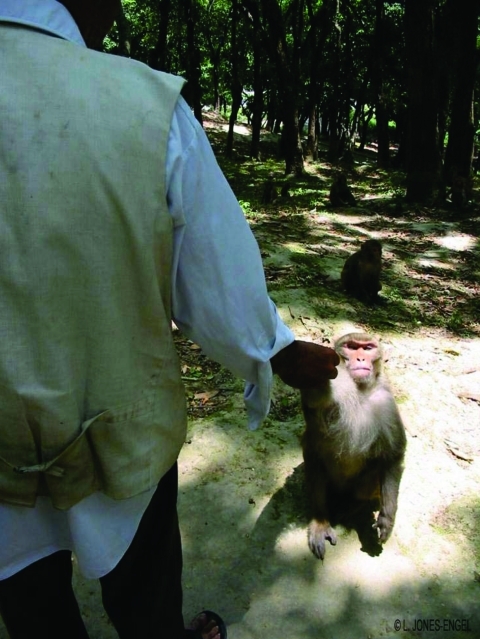
Rhesus macaques at Swoyambhu Temple routinely get food handouts from local inhabitants and visitors. (Photo by L. Jones-Engel.)

Swoyambhu Temple is 1 of 2 temple sites in the densely populated Kathmandu valley with a large population of free-ranging rhesus monkeys (*6*) (Figure 1). As 1 of the region's oldest and most important Buddhist holy places, Swoyambhu has been designated a world heritage site and continues to play a vibrant role in Kathmandu's cultural life. In addition to the Tibetan monks, Brahmin priests, and Newar nuns who live on the site, a brisk flow of local worshipers and visitors from around the world passes through Swoyambhu. Persons who live and work in and around Swoyambhu share common water sources with the macaques and report that the macaques frequently invade their homes and gardens in search of food. The macaques at Swoyambhu have become a tourist attraction in their own right, and many visitors interact with the monkeys, often by feeding or teasing them (Figure 2). A growing literature documents human-macaque interactions at monkey temples in Asia (*2**–**5*). Macaques climb on the heads and shoulders of visitors, which may bring macaque body fluids into contact with visitors' eyes and nasal and oral mucosa, potential portals of entry for infectious agents. Visitors may also be bitten or scratched by macaques during aggressive encounters, resulting in transcutaneous exposure to infectious agents present in macaque body fluids.

## Enzootic Simian Viruses

### *Cercopithecine herpesvirus* 1

*Cercopithecine herpesvirus* 1 (CHV-1), also known as herpes B virus, is a member of the taxonomic subfamily *Alphaherpesviridae*. Serologic evidence of infection with CHV-1 has been documented in several species of macaques (*7*). Seroprevalence of anti-herpesvirus antibodies is 10%–80% among wild populations and can reach 100% among captive populations, though the percentage of infected monkeys who shed virus at a given time is only 1%–2% (*8*).

While CHV-1 infection in primates is almost always benign, CHV-1 infection in humans causes severe meningoencephalitis with a death rate approaching 70% (*9*). Several routes of primate-to-human transmission have been implicated, most involving direct exposure to tissue or fluid from an infected macaque. One case of human-to-human transmission of CHV-1 has been documented (*10*). No cases of CHV-1 infection have been documented in persons exposed to free-ranging macaques, in spite of a long history of human-macaque commensalism in Asia.

### SV40

Simian virus 40 (SV40) is a polyomavirus enzootic among some species of Asian macaques, including rhesus macaques of northern India and Nepal. SV40 is present in the genitourinary tract of infected macaques and is thought to be transmitted through ingestion of urine containing the virus (*11*). SV40 first became an object of public health interest in the 1960s when millions of doses of polio vaccine, produced in tissue cultures of monkey kidney cells, were contaminated with SV40. Shah (*12*) examined the seroprevalence of antibodies to polyomavirus among workers at 2 monkey export firms in India who had abundant, long-term contact with rhesus macaques; he found a seroprevalence of 27% among these workers and noted that SV40 seroprevalence increased with duration of work in the export firms. Recent technological advances have improved the specificity of immunoassays that detect antibodies to SV40 (*13*). Using these new methods, Engels and colleagues reported evidence of human SV40 infection among zoo employees who worked with primates (*14*).

### Rhesus Cytomegalovirus

Rhesus cytomegalovirus (RhCMV), *Cercopithecine herpesvirus* 8, is a β-herpesvirus enzootic among *M. mulatta*, infecting up to 100% of rhesus macaques >1 year of age in breeding populations of captive animals (*15*). In immunologically intact animals, RhCMV infections are asymptomatic. RhCMV can cause illness and death in rhesus macaques co-infected with immunosuppressive retroviruses (simian type D retrovirus and simian immunodeficiency virus) (*16*) or in experimentally infected rhesus macaque fetuses (*17*). Infection is lifelong, with continued viral shedding from mucosal surfaces (*18*). Though growth of RhCMV in human cells has been demonstrated in vitro, human infection with RhCMV has yet to be reported (*19*).

## Enzootic Simian Retroviruses

Macaques harbor several enzootic retroviruses, including simian foamy virus (SFV), simian type D retrovirus (SRV), and simian T-cell lymphotropic virus (STLV). SRV and SFV are present in saliva and other body fluids of infected macaques, which suggests that bites, scratches, and mucosal splashes with macaque body fluids can transmit infection (*20**,**21*). Previous studies examining laboratory and zoo workers as well as bushmeat hunters in Africa and monkey temple workers in Indonesia have shown that humans can be infected with SFV and SRV (*5**,**22**–**24*).

STLV is closely related to human T-cell lymphotropic virus (HTLV-1). Asymptomatic infection with STLV-1 is common among primate hosts. STLV is hypothesized to be the progenitor of HTLV through multiple cross-species transmissions (*25*).

Simian immunodeficiency virus (SIV) is widely distributed among African primates and has been shown to infect humans who come into contact with them (*26*). Though SIV has not, to date, been detected among Asian primates, several species of Asian macaques have been experimentally infected with the virus (*27*). And though international trade in primates is regulated by the Convention on International Trade in Endangered Species, illicit import and export of primates continues, potentially exposing Asian primates to infectious agents, such as SIV, that are enzootic among African primate species.

## Materials and Methods

### Macaque Population

**Figure 3:**
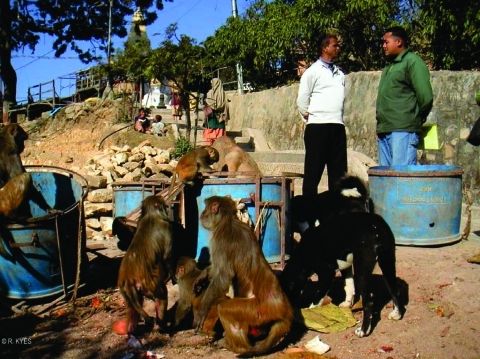
Natural forage is extremely limited at Swoyambhu. Rhesus macaques routinely raid garbage bins and people's homes in search of food. (Photo by R. Kyes.)

The rhesus macaques at Swoyambhu number ≈400, distributed among 5 to 7 groups with overlapping home ranges (*6*). Physical contact among macaque groups is common. Natural forage is extremely limited at Swoyambhu (Figure 3). Almost all of the macaques' daily food comes from handouts given by persons who frequent the temple site.

### Field Methods

During a 4-day period in May 2003, a total of 39 macaques from 3 different groups (12 from group 1, 11 from group 2, and 16 from group 3) were trapped, sampled, and released. Samples were obtained as part of a comprehensive health screening effort conducted at the request of the Federation of Swoyambhu Management and Conservation Committee. Macaques were trapped in a portable cage measuring 2.5 × 2.5 × 1.5 m and sedated with 3 mg/kg intramuscular tiletamine HCl/zolazepam HCl. To avoid stressing young animals, infants were not anaesthetized or sampled as part of this protocol. All anesthetized macaques were given a complete physical examination, and using universal precautions and sterile technique, we collected 10 mL blood by venipuncture of the femoral vein; 8 mL blood was centrifuged to extract serum. The remaining blood was aliquotted into Vacutainer vials containing EDTA. Unique study identification numbers were assigned to all specimens collected from each animal. Serum and whole blood were frozen in the field, then stored at –70°C. Animals were tattooed on their inner right thigh for identification and future follow-up. Each macaque's weight and dental formula were collected and recorded for age assessment. Age was estimated on the basis of observed dental eruption sequence. After sample collection, animals were placed in a cage and allowed to recover fully from anesthesia before being released as a group back into their home range. This data collection protocol was reviewed and approved by the University of Washington Institutional Animal Care and Use Committee (#3143-03).

### Laboratory and Data Analysis Methods

After necessary national and international permits were obtained, the samples were shipped to the United States, where they were analyzed at several institutions. Enzyme immunoassays for anticapsid antibodies to SV40 were performed as described previously (*13*). Serologic status to RhCMV was determined by enzyme-linked immunosorbent assay (ELISA) with an infected cell extract to detect RhCMV-specific immunoglobulin G (IgG) (*28*). ELISAs were used to detect antibodies to SRV, STLV, SIV, and CHV-1 as previously described (*29**,**30*). Because of endogenous seroreactivity to retroviral proteins in macaques, immunoblot assays for STLV and SRV serotypes 1, 2, 4, and 5 and were performed on all samples to confirm antibody status. Reactions were deemed positive if core and envelope bands were present, indeterminate if only core or only envelope were present, and negative if bands did not appear or were lighter than those for negative control plasma. Real-time polymerase chain reaction (PCR) for SRV was performed as previously described (*30*). To produce large volumes of SRV-1, 2, 4, and 5 antigens for enzyme immunoassay, infected cell supernatants were collected, concentrated, and purified on sucrose gradients as previously described (*29*). Nested PCR to detect STLV in these samples was performed as previously described (*30*). Western blot immunoassays for SFV were performed as previously described, with a few modifications (*5**,**31*).

Demographic and serologic data were entered into a spreadsheet, and univariate analysis was performed with the JUMP-IN 4 statistical software package (SAS Institute, Inc., Cary, NC, USA). Statistical associations between macaque viral seropositivity, sex, age class, and group number were determined by χ^2^ test.

## Results

Table 1 presents the demographic distribution of the macaques sampled. The animals sampled may not reflect the demographic breakdown of the Swoyambhu population as a whole because animals were trapped opportunistically, and infants were excluded from the study. Approximately 9.75% of Swoyambhu's estimated macaque population was sampled. Table 2 presents seroprevalence data for the 39 macaques sampled. Seven samples reacted to SRV on ELISA; 4 of these 7 were indeterminate on immunoblot, and 3 were negative. Repeated attempts to amplify SRV from all samples, including those indeterminate by immunoblot, by using PCR primers in 2 different regions of the genome were not successful. STLV testing showed 9 samples to be reactive on ELISA, but none were confirmed positive by immunoblot. Nested PCR did not detect STLV DNA in peripheral blood mononuclear cells. Additional tests for SRV and STLV by PCR were performed to rule out latent virus in genomic DNA, since some apparent false reactivity was seen on ELISA and Western blot. None of the 39 serum samples was reactive on SIV ELISA.

**Table 1:** Demographic distribution of rhesus macaques sampled at Swoyambhu

Age class	n	Males	Females
Juvenile	13	6	7
Subadult	7	1	6
Adult	19	10	9
Total	39	17	22

**Table 2:** Seroprevalence of select enzootic simian viruses among Swoyambhu rhesus macaques*†

Characteristic	n	RhCMV (% ELISA-reactive)	SV40 (% EIA-reactive)	CHV-1 (% ELISA-reactive)	SFV (% WB-reactive)
Male	17	94.1	94.1	64.7	94.1
Female	22	95.5	86.4	63.6	100.0
Juvenile	13	84.6	76.9	23.1	92.3
Subadult	7	100.0	100.0	42.9	100.0
Adult	19	100.0	94.7	100.0	100.0
Total	39	94.9	89.7	64.1	97.4

*RhCMV, rhesus cytomegalovirus; ELISA, enzyme-linked immunosorbent assay; SV40, simian virus 40; EIA, enzyme immunoassay; CHV-1, cercopithecine herpesvirus 1; SFV, simian foamy virus; WB, Western blot. †Seven samples were ELISA-positive for simian retrovirus (SRV); 4 of these were indeterminate on WB, and 3 were negative. Polymerase chain reaction (PCR) failed to amplify SRV from any sample. Nine samples were ELISA-positive for simian T-cell lymphotropic virus (STLV), but none were positive on immunoblot, and nested PCR detected no STLV DNA. None of the samples was reactive to simian immunodeficiency virus.

The results of the serologic and PCR assays were analyzed by using χ^2^ to test associations by sex, group number, and age category. The results from these tests show no significant association between seropositivity for antibodies to RhCMV, SV40, or SFV and age, sex, or group number of the macaque. However, a significant (χ^2^ p<0.0001) age-related effect was seen for CHV-1. Seroprevalence of antibodies to CHV-1 increased from 23.1% (3/13) among juveniles to 100% (19/19) among adult macaques.

## Discussion

Relatively little is known about enzootic primate viruses in free-ranging populations of macaques. CHV-1 antibody prevalence has been measured among temple monkeys in Bali (*4*), rhesus monkeys (*M. mulatta)* from India (*32*), and free-ranging rhesus monkeys transplanted to the Caribbean Island of Cayo Santiago (*11*). The CHV-1 seroprevalence among these populations is similar to that of the Swoyambhu macaques, with a similar positive association between age and seroprevalence. SFV prevalence among the Bali macaques was also similar to that measured in the Swoyambhu macaques (*5*). The high seroprevalence of RhCMV in the Swoyambhu macaque population mirrors that measured in other studies that assessed seroprevalence in both captive and free-ranging populations of macaques and other primates (*15**,**33*).

Evidence of STLV-1 infection was not found among the Swoyambhu macaques with either serologic or PCR detection methods. In comparison, a survey measuring STLV-1 prevalence among wild-caught *M. fascicularis* in Indonesia by serologic methods and PCR suggested an STLV prevalence between 3.3% and 10% (*34*), and a sample of wild-caught *M. fascicularis* from 9 localities in Thailand were all antibody-negative for STLV-1 (*35*).

No conclusive serologic or PCR evidence of SRV infection was found among the Swoyambhu macaques. SRV infection is commonly seen among laboratory primates, but far less so among other free-ranging primate populations examined to date (L. Jones-Engel, unpub. data). Increased population densities characteristic of captive settings may facilitate viral transmission, providing a possible explanation for this observation.

The absence of SIV in the Swoyambhu macaque population is unsurprising, given that SIV has yet to be detected in natural populations of Asian primates. This finding, however, does not eliminate the possibility that the situation could change in the future. While SIV is typically found only among African primates, this virus could be introduced into Asian primate populations through the global market trade in animals, in which pet primates can be purchased (*36*). Abandoned pet primates are commonly seen in monkey forests in Asia, and future surveys of Asian primates should continue to test for SIV.

### Public Health Implications

A growing literature suggests that cross-species transmission of infectious agents occurs between humans and several primate species in a variety of contexts and in diverse areas (*4**,**5**,**22**–**24**,**37**,**38*). Indeed, wherever humans and primates come into contact, the potential for cross-species transmission exists. Whether cross-species transmission occurs depends on several factors, including the prevalence of infectious agents in primate reservoirs, the context of interspecies contact, and the frequency with which contact occurs (*39*). To date, cross-species transmission has been most thoroughly studied in primate laboratories and zoos because of the ready availability of biological samples from both primates and exposed humans (*14**,**22**,**23**,**40*). Research on humans exposed to primates in these contexts has documented an SFV seroconversion rate of 1% to 5.3%, an SV40 seroconversion rate of 3% to 10%, and an SRV rate of 0.9%. Human CHV-1 infection is rare and has only been documented among persons directly or indirectly exposed to laboratory primates (*9*).

The dynamics of human-macaque contact at Asian monkey temples differ substantially from those in laboratories and zoos, which may make cross-species transmission more likely at monkey temples. Because primate laboratories have been promoting specific pathogen-free colonies, the risk for primate-to-human transmission of enzootic agents in these settings is likely to diminish over time. Additionally, primate laboratories require the routine use of eye protection, gloves, and protective garments. Injury protocols call for thorough irrigation of wounds and close follow-up of exposed persons. In contrast, research examining wound care practices among exposed workers at the Sangeh monkey forest in Bali (*4*) found no use of protective eyewear, gloves, or protective clothing. Bleeding wounds from macaque scratches and bites were often not cleansed, and only 6 of 51 persons bitten or scratched by a macaque sought medical care (*4*). As a result, exposure to bites, scratches, and mucosal splashes at monkey temples may carry a higher risk for primate-to-human viral transmission than does exposure in primate laboratories and zoos.

From a global infection control standpoint, learning about primate-to-human transmission at monkey temples like Swoyambhu is particularly relevant. Because the number of humans who come into contact with primates at monkey temples around the world is probably several million per year (*3*), monkey temples are an important interface between humans and primates. Additionally, many of the visitors to Swoyambhu are foreign tourists, which makes Swoyambhu a potential point source for the global dispersal of infectious agents, as world travelers can return to their homes carrying novel infectious agents transmitted from macaques. Monkey temples of South and Southeast Asia are also near large human population centers. The combination creates the potential for rapid global dispersal of primateborne infectious agents to human populations around the world.

In spite of centuries of human-primate commensalism in Asia, human disease has yet to be causally linked to enzootic primateborne viruses. However, disease may go undetected because of low incidence, inadequate surveillance, and lack of awareness. Latency between infection and disease manifestation could mask the association between primate exposure and disease. Though long-term commensalism may lead to increased immunity to primateborne pathogens among exposed human populations, non-Asian visitors might be vulnerable, and increased travel to Asia would expose more nonimmune persons to primateborne pathogens. Finally, as HIV continues to spread in Asia, increasing numbers of immunosuppressed persons will likely be exposed to primateborne infectious agents. Immunocompromised hosts could provide pathogens a "permissive" environment in which to evolve into more pathogenic forms.

### Management Strategies

In taking steps to reduce the risk for cross-species transmission between humans and primates, we should first understand how transmission occurs, i.e., the situations, conditions, and behavior that lead to contact and transmission. Because feeding macaques is thought to account for most interactions between humans and macaques, adopting protocols to restrict feeding to persons specifically trained for that purpose could reduce the number of visitors who come into direct contact with macaque body fluids. Educating visitors as well as persons who live near monkey temples to avoid behavior that leads to bites and scratches could reduce risk. Finally, availability and awareness of proper protocol for effective wound irrigation has the potential to reduce transmission of infection. No data on the efficacy of postexposure prophylaxis with antiviral agents are available, but pharmacologic therapy is another tool that could reduce the likelihood of cross-species viral transmission. In addition, monitoring human populations for infection with primate viruses at the human-primate interface is a prudent strategy to facilitate the early detection of primateborne zoonoses.

This information must be put into context. The recent culling of macaques at a wildlife park in England and at monkey temples in Hong Kong and Taiwan in response to the perceived threat of zoonotic transmission of CHV-1 is an example of an exaggerated response to an inadequately understood risk. Improving awareness of zoonotic transmission and effective management strategies among the public as well as among persons who manage primate parks and temples will be instrumental in allowing humans and primates to continue to coexist.

## References

[1] Cleaveland S, Laurenson MK, Taylor LH. Diseases of humans and their domestic mammals: pathogen characteristics, host range and the risk of emergence. Philos Trans R Soc Lond B Biol Sci. 2001;356:991–9. 10.1098/rstb.2001.0889 11516377 PMC1088494

[2] Fuentes A, Gamerl S. Disproportionate participation by age/sex classes in aggressive interactions between long-tailed macaques (*Macaca fascicularis*) and human tourists at Padangtegal Monkey Forest in Bali, Indonesia. Am J Primatol. 2005;66:197–204. 10.1002/ajp.20138 15940713

[3] Fuentes A, Southern M, Suaryana KG. Monkey forests and human landscapes: is extensive sympatry sustainable for *Homo sapiens* and *Macaca fascicularis* on Bali? In: Patterson J, Wallis J, editors. Commensalism and conflict: the human-primate interface. Norman (OK): ASP Press; 2005. p. 165–95.

[4] Engel GA, Jones-Engel L, Schillaci MA, Suaryana KG, Putra A, Fuentes A, Human exposure to herpesvirus B-seropositive macaques, Bali, Indonesia. Emerg Infect Dis. 2002;8:789–95. 12141963 10.3201/eid0808.010467 PMC3266706

[5] Jones-Engel L, Engel G, Schillaci M, Rompis A, Putra A, Suaryana KG, Primate-to-human retroviral transmission in Asia. Emerg Infect Dis. 2005;11:1028–35. 16022776 10.3201/eid1107.040957 PMC3371821

[6] Chalise MK, Ghimire M. Non-human primate census in different parts of Nepal. Bulletin of the Natural History Society of Nepal. 1998;8:11–5.

[7] Kalter SS, Heberling RL, Cooke AW, Barry JD, Tian PY, Northam WJ. Viral infections of nonhuman primates. Lab Anim Sci. 1997;47:461–7. 9355086

[8] Kessler MJ, Hilliard JK. Seroprevalence of B virus (*Herpesvirus simiae*) antibodies in a naturally formed group of rhesus macaques. J Med Primatol. 1990;19:155–60. 2160017

[9] Huff JL, Barry PA. B-virus (cercopithecine herpesvirus 1) infection in humans and macaques: potential for zoonotic disease. Emerg Infect Dis. 2003;9:246–50. 12603998 10.3201/eid0902.020272 PMC2901951

[10] Centers for Disease Control and Prevention. B-virus infection in humans—Pensacola, Florida. MMWR Morb Mortal Wkly Rep. 1987;36:289–90. 3033462

[11] Shah KV, Morrison JA. Comparison of three rhesus groups for antibody patterns to some viruses: absence of active simian virus 40 transmission in the free-ranging rhesus of Cayo Santiago. Am J Epidemiol. 1969;89:308–15. 4304325 10.1093/oxfordjournals.aje.a120943

[12] Shah KV. Neutralizing antibodies to simian virus 40 (SV40) in human sera from India. Proc Soc Exp Biol Med. 1966;121:303–7. 4285770 10.3181/00379727-121-30765

[13] Viscidi RP, Rollison DE, Viscidi E, Clayman B, Rubalcaba E, Daniel R, Serological cross-reactivities between antibodies to simian virus 40, BK virus, and JC virus assessed by virus-like-particle-based enzyme immunoassays. Clin Diagn Lab Immunol. 2003;10:278–85. 12626455 10.1128/CDLI.10.2.278-285.2003 PMC150538

[14] Engels EA, Switzer WM, Heneine W, Viscidi RP. Serologic evidence for exposure to simian virus 40 in North American zoo workers. J Infect Dis. 2004;190:2065–9. 10.1086/425997 15551203

[15] Vogel P, Weigler BJ, Kerr H, Hendrickx A, Barry PA. Seroepidemiologic studies of cytomegalovirus infection in a breeding population of rhesus macaques. Lab Anim Sci. 1994;44:25–30. 8007656

[16] Kaur A, Kassis N, Hale CL, Simon M, Elliott M, Gomez-Yafal A, Direct relationship between suppression of virus-specific immunity and ergence of cytomegalovirus disease in simian AIDS. J Virol. 2003;77:5749–58. 10.1128/JVI.77.10.5749-5758.2003 12719568 PMC154043

[17] Chang WL, Tarantal AF, Zhou SS, Borowsky AD, Barry PA. A recombinant rhesus cytomegalovirus expressing enhanced green fluorescent protein retains the wild-type phenotype and pathogenicity in fetal macaques. J Virol. 2002;76:9493–504. 10.1128/JVI.76.18.9493-9504.2002 12186931 PMC136446

[18] Huff JL, Eberle R, Capitanio J, Zhou SS, Barry PA. Differential detection of B virus and rhesus cytomegalovirus in rhesus macaques. J Gen Virol. 2003;84:83–92. 10.1099/vir.0.18808-0 12533703

[19] Alcendor DJ, Barry PA, Pratt-Lowe E, Luciw PA. Analysis of the rhesus cytomegalovirus immediate-early gene promoter. Virology. 1993;194:815–21. 10.1006/viro.1993.1323 8389084

[20] Lerche NW, Osborn KG, Marx PA, Prahalada S, Maul DH, Lowenstine LJ, Inapparent carriers of simian acquired immune deficiency syndrome type D retrovirus and disease transmission with saliva. J Natl Cancer Inst. 1986;77:489–96. 3461210

[21] Murray SM, Picker LJ, Axthelm MK, Linial ML. Expanded tissue targets for foamy virus replication with simian immunodeficiency virus–induced immunosuppression. J Virol. 2006;80:663–70. 10.1128/JVI.80.2.663-670.2006 16378969 PMC1346877

[22] Lerche NW, Switzer WM, Yee JL, Shanmugam V, Rosenthal AN, Chapman LE, Evidence of infection with simian type D retrovirus in persons occupationally exposed to nonhuman primates. J Virol. 2001;75:1783–9. 10.1128/JVI.75.4.1783-1789.2001 11160676 PMC114087

[23] Switzer WM, Bhullar V, Shanmugam V, Cong ME, Parekh B, Lerche NW, Frequent simian foamy virus infection in persons occupationally exposed to nonhuman primates. J Virol. 2004;78:2780–9. 10.1128/JVI.78.6.2780-2789.2004 14990698 PMC353775

[24] Wolfe ND, Switzer WM, Carr JK, Bhullar VB, Shanmugam V, Tamoufe U, Naturally acquired simian retrovirus infections in central African hunters. Lancet. 2004;363:932–7. 10.1016/S0140-6736(04)15787-5 15043960

[25] Vandamme AM, Salemi M, Desmyter J. The simian origins of the pathogenic human T-cell lymphotropic virus type I. Trends Microbiol. 1998;6:477–83. 10.1016/S0966-842X(98)01406-1 10036726

[26] Hahn BH, Shaw GM, De Cock KM, Sharp PM. AIDS as a zoonosis: scientific and public health implications. Science. 2000;287:607–14. 10.1126/science.287.5453.607 10649986

[27] Allan JS, Short M, Taylor ME, Su S, Hirsch VM, Johnson PR, Species-specific diversity among simian immunodeficiency viruses from African green monkeys. J Virol. 1991;65:2816–28. 2033656 10.1128/jvi.65.6.2816-2828.1991 PMC240900

[28] Yue Y, Zhou SS, Barry PA. Antibody responses to rhesus cytomegalovirus glycoprotein B in naturally infected rhesus macaques. J Gen Virol. 2003;84:3371–9. 10.1099/vir.0.19508-0 14645918

[29] Kuller L, Watanabe R, Anderson D, Grant R. Development of a whole virus multiplex flow cytometric assay for rapid screening of specific pathogen-free primate colony. Diagn Microbiol Infect Dis. 2005;53:185–93. 10.1016/j.diagmicrobio.2005.05.012 16243475

[30] Schillaci MA, Jones-Engel L, Engel GA, Paramastri Y, Iskandar E, Wilson B, Prevalence of enzootic simian viruses among urban performance monkeys in Indonesia. Trop Med Int Health. 2005;10:1305–14. 10.1111/j.1365-3156.2005.01524.x 16359412

[31] Khan AS, Sears JF, Muller J, Galvin TA, Shahabuddin M. Sensitive assays for isolation and detection of simian foamy retroviruses. J Clin Microbiol. 1999;37:2678–86. 10405421 10.1128/jcm.37.8.2678-2686.1999 PMC85313

[32] Orcutt RP, Pucak GJ, Foster HL, Kilcourse JY. Multiple testing for the detection of B virus antibody in specially handled rhesus monkeys after capture from virgin trapping grounds. Lab Anim Sci. 1976;26:70–4. 177807

[33] Blewett EL, Lewis J, Gadsby EL, Neubauer SR, Eberle R. Isolation of cytomegalovirus and foamy virus from the drill monkey (*Mandrillus leucophaeus*) and prevalence of antibodies to these viruses amongst wild-born and captive-bred individuals. Arch Virol. 2003;148:423–33. 10.1007/s00705-002-0937-9 12607096

[34] Richards AL, Giri A, Iskandriati D, Pamungkas J, Sie A, Rosen L, Simian T-lymphotropic virus type I infection among wild-caught Indonesian pig-tailed macaques (*Macaca nemestrina*). J Acquir Immune Defic Syndr Hum Retrovirol. 1998;19:542–5. 10.1097/00042560-199812150-00015 9859970

[35] Ishida T, Varavudhi P. Wild long-tailed macaques (*Macaca fascicularis*) in Thailand are highly infected with gamma herpes virus but not with simian T-lymphotropic retrovirus of type 1. Folia Primatol (Basel). 1992;59:163–8. 10.1159/000156654 1339088

[36] Karesh WB, Cook RA, Bennett EL, Newcomb J. Wildlife trade and global disease emergence. Emerg Infect Dis. 2005;11:1000–2. 16022772 10.3201/eid1107.050194 PMC3371803

[37] Jones-Engel L, Engel G, Schillaci M, Lee B, Heidrich J, Chalise M, Considering human to primate transmission of measles virus through the prism of risk analysis. Am J Primatol. 2006. In press. 10.1002/ajp.20294 16900498

[38] Jones-Engel L, Engel GA, Schillaci MA, Babo R, Froehlich J. Detection of antibodies to selected human pathogens among wild and pet macaques (*Macaca tonkeana*) in Sulawesi, Indonesia. Am J Primatol. 2001;54:171–8. 10.1002/ajp.1021 11443632

[39] Engel G, Hungerford L, Jones-Engel L, Travis D, Fuentes A, Grant R, Risk assessment: a model for predicting cross-species transmission of SFV from macaques (*M. fascicularis*) to humans at a monkey temple in Bali, Indonesia. Am J Primatol. 2006. In press. 10.1002/ajp.20299 16900504

[40] Sandstrom PA, Phan KO, Switzer WM, Fredeking T, Chapman L, Heneine W, Simian foamy virus infection among zoo keepers. Lancet. 2000;355:551–2. 10.1016/S0140-6736(99)05292-7 10683011

